# Identification of the safe(r) by design alternatives for nanosilver-enabled wound dressings

**DOI:** 10.3389/fbioe.2022.987650

**Published:** 2022-10-11

**Authors:** V. Cazzagon, E. Giubilato, A. Bonetto, M. Blosi, I. Zanoni, A. L. Costa, C. Vineis, A. Varesano, A. Marcomini, D. Hristozov, E. Semenzin, E. Badetti

**Affiliations:** ^1^ Department of Environmental Sciences Informatics and Statistics, Ca’ Foscari University of Venice, Venice Mestre, Italy; ^2^ GreenDecision Srl, Venice Mestre, Italy; ^3^ Institute of Science and Technology for Ceramics (CNR-ISTEC), National Research Council of Italy, Faenza, Italy; ^4^ Institute of Intelligent Industrial Technologies and Systems for Advanced Manufacturing (CNR-STIIMA), National Research Council of Italy, Biella, Italy

**Keywords:** silver, wound dressing, analytical chemistry, safe-by-design, nanomaterials, medical device, antimicrobial activity

## Abstract

The use of silver nanoparticles (NPs) in medical devices is constantly increasing due to their excellent antimicrobial properties. In wound dressings, Ag NPs are commonly added in large excess to exert a long-term and constant antimicrobial effect, provoking an instantaneous release of Ag ions during their use or the persistence of unused NPs in the wound dressing that can cause a release of Ag during the end-of-life of the product. For this reason, a Safe-by-Design procedure has been developed to reduce potential environmental risks while optimizing functionality and costs of wound dressings containing Ag NPs. The SbD procedure is based on ad-hoc criteria (e.g., mechanical strength, antibacterial effect, leaching of Ag from the product immersed in environmental media) and permits to identify the best one among five pre-market alternatives. A ranking of the SbD alternatives was obtained and the safer solution was selected based on the selected SbD criteria. The SbD framework was also applied to commercial wound dressings to compare the SbD alternatives with products already on the market. The iterative procedure permitted to exclude one of the alternatives (based on its low mechanical strength) and proved to be an effective approach that can be replicated to support the ranking, prioritisation, and selection of the most promising options early in the innovation process of nano-enabled medical devices as well as to encourage the production of medical devices safer for the environment.

## 1 Introduction

The use of silver nanoparticles (Ag NPs) in medical devices (e.g., coatings on implants, catheters and medical bandages) (SCENIHR, 2015) is constantly increasing due to the well-known antimicrobial properties of Ag (António et al., 2015). In this context, the antimicrobial action of wound dressings (WDs) containing Ag NPs is mainly related to the release of Ag in the ionic form (Nešporová et al., 2020), which can interact with components of the bacteria cells, reducing respiration and provoking their subsequent inactivation and lysis (Yetisen et al., 2016; Musino et al., 2021). Moreover, as the Ag NPs are added in WDs in high concentration to exert a long-term and constant antimicrobial effect, an excessive instantaneous release of Ag^+^ or the persistence of unused Ag NPs can be observed (Musino et al., 2021).

The antimicrobial action of the nanoscale Ag is a desired functionality, but it is important to ensure that the Ag-based WDs are also safe for the patients and the environment.

To this end, the Ag content used in WDs needs to be properly evaluated adopting a life cycle perspective, by investigating not only the safety aspects during their use (i.e., the application of the wound dressing on the injured skin), but also in the end-of-life, through the assessment of the Ag released in environmental compartments and its effects. Indeed, Ag NPs used in WDs can potentially be released and reach wastewater treatment plants where the Ag can be retained in sewage sludge, which could be then used as fertilizer for agricultural soils. Through runoff water, Ag NPs can reach aquatic environments (McGillicuddy et al., 2017; Zhu et al., 2019) leading to lethal effects for representative species of aquatic plants, invertebrates and fishes (SCENIHR, 2014). In the soil organism *E. crypticus*, Ag concentrations in organisms exposed to Ag NPs keep increasing for longer time than in organisms exposed to AgNO_3_, leading to a higher risk of longer-term exposure of Ag NPs compared to Ag^+^ (Santos et al., 2021). Moreover, Ag can interact with sulphur that naturally exists in anaerobic environments to form Ag_2_S in soil, or with Cl^−^ forming AgCl in aquatic environments (Zhang et al., 2018), potentially provoking additional effects on aquatic and terrestrial organisms.

To reduce the potential health and environmental risks posed by these NPs, it is relevant to implement Safe-by-Design (SbD) approaches that aim at controlling the release of Ag into the environment, as already investigated in the case of antimicrobial ceramic tiles (Gardini et al., 2018). To study the release of the Ag from the WDs to (compromised) skin as well as into relevant environmental compartments (i.e., surface water, soil) targeted leaching tests can be performed.

The SbD concept has been widely applied to nano-enabled products in several sectors such as the conservation of works of art (Semenzin et al., 2019), paints, biosensors or automotive applications (Sánchez Jiménez et al., 2020), smart nanomaterials used in agriculture, food, food packaging and cosmetics (Gottardo et al., 2021) and nanomedicines (Schmutz et al., 2020). The application of this concept involves identification of material/product design alternatives already at the early stage of the innovation process to reduce the potential for release of hazardous chemicals/materials and/or decrease their biopersistence, bioaccumulation and/or hazard, while retaining functionality for their intended uses (Schmutz et al., 2020; Soeteman-Hernández et al., 2020).

In this manuscript we propose a SbD procedure aimed at guiding the selection of alternatives among five WDs containing Ag NPs based on criteria pertaining to safety, functionality, and cost. It is based on the framework for risk management of nano-biomaterials (NBMs) used in medical devices developed in the H2020 BIORIMA project (Giubilato et al., 2020) and will be extended considering sustainability aspects in the context of the H2020 SUNSHINE project (G.A. 952924). As wound dressings containing Ag NPs (indicated as Ag-WDs hereafter) should have a sufficient antimicrobial effect but simultaneously could release significative amount of Ag into the environment, a trade-off between the factors affecting the performance and the safety of Ag-WDs should be pursued. In this regard, considerations and functionality of the selected wound dressings are investigated and *ad-hoc* experimental tests have been selected and used as criteria associated to the three SbD objectives of this study: 1) maximisation of the antimicrobial activity of the Ag-WD, 2) reduction of possible Ag released into the environment, 3) optimization of the cost-effectiveness of the Ag-WD.

To this purpose, a physico-chemical characterization and leaching tests were performed to support the SbD procedure. Specifically, morphological analysis of the wound dressings using Scanning Electron Microscopy (SEM) and a quantification of Ag in Ag-WDs using Inductively Coupled Plasma-Mass Spectrometry (ICP-MS) were conducted. Moreover, leaching of Ag from each Ag-WD immersed in synthetic sweat (to simulate the use stage) and environmental media (to simulate the end-of-life stage) was also assessed using ICP-MS. Finally, to assess the antimicrobial efficacy of Ag-WDs, antimicrobial tests were performed against *E. coli*.

## 2 Materials and methods

### 2.1 Procedure to prioritize SbD alternatives

The SbD procedure proposed in this manuscript is based on a thorough analysis of the state-of-the-art literature. To identify relevant publications, a literature search was conducted, covering both SbD approaches applied specifically to nano and biomaterials used in medical devices and/or medicinal products, or to nano-enabled applications in general. The focus was specifically on papers including information about WDs containing NPs. The literature search identified more than 40 relevant publications from both the grey and peer-reviewed literature, which were carefully analysed. Based on this analysis, relevant criteria were identified to assess the functionality, costs and environmental safety of WDs containing NPs (Kraegeloh et al., 2018).

The proposed SbD approach covers the full lifecycle of the WDs and particularly focuses on the end-of-life as suggested by Tavernaro et al., 2021. Our methodology is also significantly influenced by the approach for nanomedicines proposed by Schmutz et al., 2020, which considers material properties and efficacy, health and environmental safety, and costs as key factors affecting SbD decision making for nanomedical applications, while important criteria such as the reduction of NPs released (Kraegeloh et al., 2018) and antibacterial activity testing (Marassi et al., 2019; Sánchez Jiménez et al., 2020) were proposed in previous studies and used in this work.

The proposed SbD approach is aimed at supporting a preliminary screening analysis for the early stages of product development to guide the selection of safer Ag-WDs alternatives that retain technical functionality for their intended uses without incurring too high costs.

Once such alternative(s) are identified, further analyses of possible human health and environmental risks should be performed before introducing the Ag-WDs to the market, but these latter analyses are beyond the scope of this work.

The SbD procedure and the identified tests are described in detail in Section 3.1.

### 2.2 Test samples

The SbD procedure has been applied to identify safer alternatives among five topical Ag-WDs, which differ in 1) the polymer used for the matrix, 2) the type and 3) the quantity of Ag NPs incorporated in the Ag-WD. Moreover, this SbD procedure has been applied to two commercial Ag-WDs, ActicoatFlex 3 and ActicoatFlex 7. In Table 1, the investigated Ag-WDs and their main characteristics are reported.

**TABLE 1:** Ag-WDs and their main characteristics.

Ag-WDs	Type of Ag	Type of Matrix	Ag Concentration on Fibres (%wt)	WD Type
PLLA-Ag	Uncoated	PLLA	4	SbD alternative
PLLA-AgHEC	HEC-coated	PLLA	5	SbD alternative
PVA-Ag	Uncoated	PVA	0.7	SbD alternative
PVA-AgHEC.1 h	HEC-coated	PVA	0.7	SbD alternative
PVA-AgHEC.2 h	HEC -coated	PVA	0.7	SbD alternative
Acticoat Flex 3	Unknown	Polyester	Unknown	Commercial WD
Acticoat Flex 7	Unknown	Polyester	Unknown	Commercial WD

More information on the main characteristics and the methods used to characterize the samples (i.e., TEM analysis) can be found in SI1.

### 2.3 Analytical techniques and methods used to test the SbD alternatives

To identify the safest Ag-WD among the five SbD alternatives and confirm that its functionality is still in commercially viable ranges, several analytical techniques and methods were applied. To assess morphological characteristics of the fibres, Scanning Electron Microscopy (SEM) images of each Ag-WDs were obtained before and after immersion in synthetic sweat by means of a JSM-6010PLUS/LA SEM. Moreover, where granules of Ag NPs or NaCl salts were visible from SEM images using a spectrometer Oxford INCA-350 Energy Dispersive X-ray (EDX) analysis was also performed in fibres to verify the presence of Ag.

To determine the total Ag content in Ag-WDs, each Ag-WD was immersed in nitric acid and ICP-MS (NexION 350D, Perkin Elmer) analyses were performed to measure the total amount of Ag. In addition, the leaching of Ag during the total immersion of the Ag-WD in synthetic sweat (simulating the worst-case scenario) was also investigated, by quantifying over time by ICP-MS the Ag released from the Ag-WDs immersed in the medium.

To estimate the amount of Ag potentially released during the end-of-life stage into environmental compartments, considering a worst-case scenario, leaching tests of Ag from the total immersion of Ag-WDs in Artificial Fresh Water (AFW), Artificial Marine Water (AMW) and soil: water extract were conducted.

As explained in the review by Brunelli et al., 2021, leaching tests can be performed according to standard protocols (International Organization for Standardization, 2018; The British Standards Institution, 2004) by a partial or a total immersion of the nano-enabled product in a liquid, followed by the quantification of NPs and/or its ionic form at different time of immersion (for a maximum of 4 weeks). The reported standard protocols have been developed for nano-based paints and paints debris. However, in these protocols the leaching of NPs is considered to determine the resistance of a nano-based material to liquids, while in the investigated medical devices NPs and ions are intentionally released from the nano-enabled product. Therefore, the above-mentioned standards protocols were opportunely modified in the current work (See paragraph 3.2.3).

The antibacterial activity of each Ag-WD was evaluated according to ASTM E 2149-01 “Standard test method for determining the antimicrobial activity of immobilized antimicrobial agents under dynamic contact conditions”. This method is designed to evaluate the resistance of antimicrobial treated specimens to the growth of microbes under dynamic contact conditions. As the focus of this manuscript is to select relevant tests to identify the safer solution among Ag-WDs at the screening level, antimicrobial efficacy was performed against only one species of bacterium, and we selected *Escherichia coli* ATCC 11229 bacterium as it is a well-studied Gram-negative species.

## 3 Results and discussion

### 3.1 SbD procedure

**FIGURE 1:**
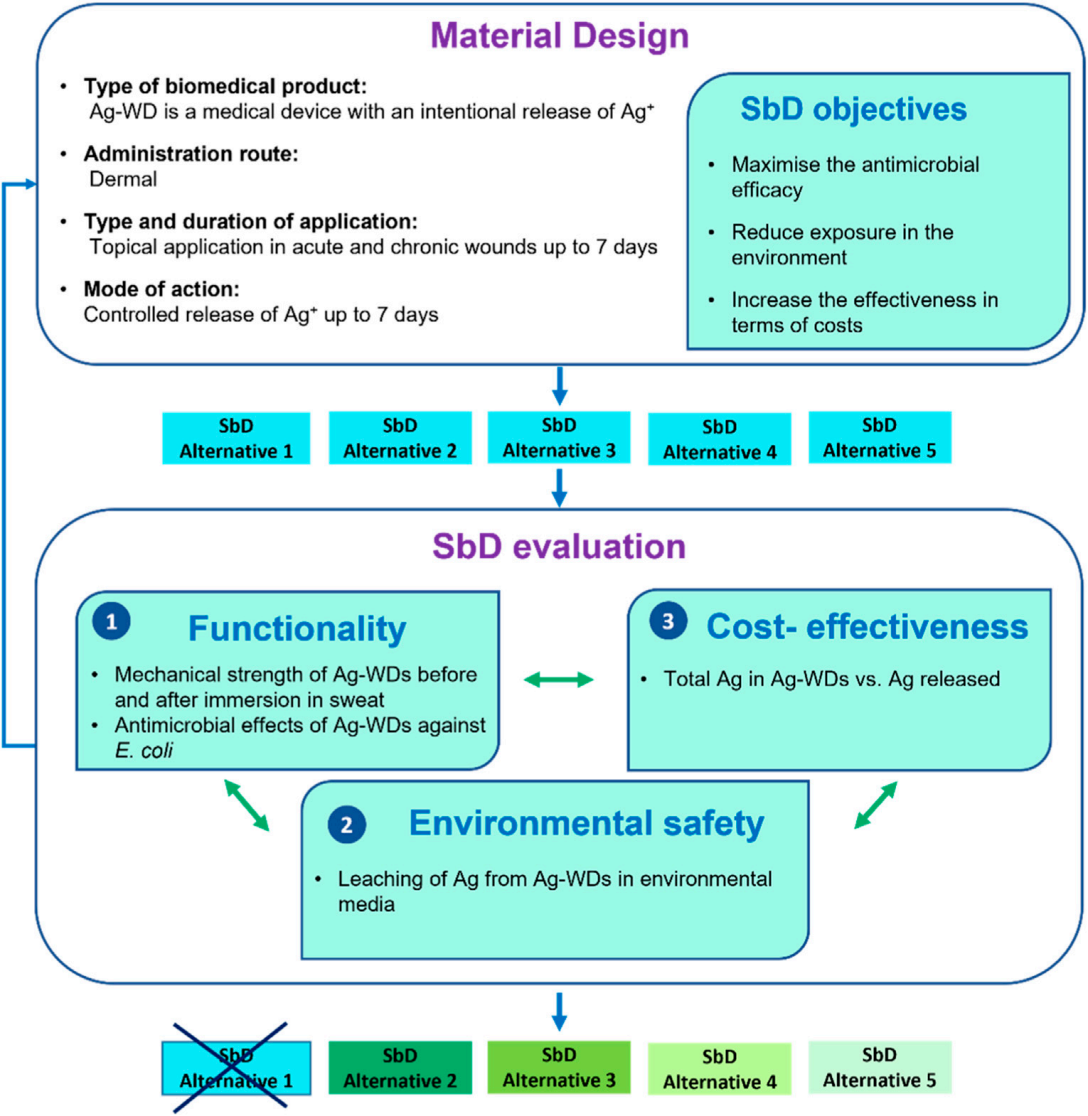
SbD procedure for topical wound dressing containing Ag NPs.

Based on the literature search, a SbD procedure was developed for WDs containing Ag NPs (Figure 1), which consists of two main pillars: Material Design and SbD evaluation.

The first step of the SbD procedure corresponds to the “Material Design”, which is guided by considerations and information about the investigated/desired product, such as its classification (i.e., medical device), the type and the duration of application (i.e., topical application up to 7 days), the administration route (i.e., dermal) and its mode of action (i.e., a controlled release of Ag^+^ up to 7 days).

During the material design, it is important to define the SbD objectives and for our case they are: 1) maximisation of the antimicrobial activity of the Ag-WD, 2) reduction of possible Ag released into the environment, 3) optimization of the cost-effectiveness of the Ag-WD. These three main objectives are interconnected, as the SbD approach requires to optimize the balance between efficacy, safety, and costs.

Once all the information on the investigated materials is assessed and SbD objectives are fixed, the SbD criteria are selected in the “SbD evaluation” step to investigate the trade-off between functionality, environmental safety, and cost-effectiveness.

To evaluate the functionality, the mechanical strength was tested through SEM analysis of the Ag-WD before and after the total immersion of the Ag-WD in synthetic sweat (see Paragraph 3.2.1). If the investigated Ag-WDs do not have a sufficient mechanical strength (i.e., shape and size of the fibres are not preserved after immersion in sweat), the material is modified (i.e., by returning to the Material Design step of the framework) or discarded (i.e., not considered in the following analysis). Moreover, since the functionality of wound dressings can be considered as the capacity of the Ag-WD to exert a sufficient antimicrobial effect, antimicrobial tests in *E. coli* were considered as second test in the SbD evaluation (Paragraph 3.2.2). If the material does not exert a sufficient antimicrobial effect, it is modified (i.e., by returning to the Material Design step of the framework) or discarded (i.e., not considered in further analysis).

Considering possible exposure in the different environmental compartments, leaching of Ag from the complete immersion of Ag-WDs were also investigated using three environmental media (i.e., freshwater, marine water, and a soil-water extract) in order to quantify Ag released in a worst-case scenario (i.e., assuming an incorrect disposal of Ag-WDs through littering on soil and water) (Paragraph 3.2.3).

Moreover, the cost-effectiveness of the Ag-WD was assessed through the ratio between the total Ag content in Ag-WDs after their total immersion in acidic conditions and leaching of Ag from Ag-WDs immersed in synthetic sweat up to 7 days (simulating the use stage), investigated by ICP-MS (see paragraph 3.2.4).

It was not possible to identify for each of the selected SbD criteria an optimal value/range to provide an absolute evaluation of each alternative, therefore the Ag-WDs were ordered according to each criterion, namely: 1) mechanical strengths, 2) antimicrobial efficacy, 2) leaching of Ag from Ag-WDs during the total immersion of Ag-WDs in environmental media, and 3) cost-effectiveness (CE) value.

For both the antimicrobial effect of Ag-WDs in *E. coli* and their cost-effectiveness, SbD alternatives were ordered from the highest to the lowest antimicrobial effect and CE % values, respectively, while for Ag leaching in environmental media, the best alternative was considered the one with the lowest value of released Ag.

### 3.2 Application of the SbD procedure: Experimental methods for each criterion

#### 3.2.1 Mechanical strengths of Ag-WDs

SEM images of each Ag-WD before and after immersion in synthetic sweat were obtained to evaluate their mechanical strength. Specifically, one piece of Ag-WD (2.5 × 2.5 cm) of each sample was totally immersed for 24 h in 10 ml of synthetic sweat, and then dried at ambient air for 2 days.

The specimens were cut and attached to an aluminium stub by a double-stick carbon tape; the specimens were also coated with a thin film of carbon (10 nm thick), using a Carbon Coater-Balzers CED-010. The resulting SEM images were assessed using ImageJ software to obtain fibres diameter reported as mean ± standard deviation (in µm) of 100 measurements of each sample.

#### 3.2.2 Antimicrobial tests of Ag-WDs against *E. coli*

The incubated test culture in a nutrient broth was diluted obtaining a concentration of 1.5–3.0 × 10^5^ CFUmL^−1^ (working dilution). Each sample (i.e., 5 × 5 cm piece of each Ag-WD) was contacted to the working dilution at the ratio 1 g of material in 50 ml of solution, based on the selected standard ASTM E 2149-01 ASTM International (2001). To evaluate the bacterial action of the dispersions against *E. coli*, the equivalent amount of Ag in WDs was calculated. All flasks were shaken for 1 h at 190 rpm. After a series of dilutions, 1 ml of the solution was plated in nutrient agar. The inoculated plates were incubated at 37°C for 24 h and surviving cells were counted. The antibacterial activity was expressed in % reduction of the organisms after contact with the test specimen compared to the number of bacterial cells surviving after contact with the control, according to Equation 1:

$$\text{Reduction }\left(\text{\%}\right)=\frac{B-A}{B}\times 100$$ (1)

where *A* is CFU mL^−1^ after contact (end test) and *B* is CFU mL^−1^ at zero contact time.

#### 3.2.3 Leaching tests of Ag from Ag-WDs during total immersion in environmental media

To estimate the amount of Ag released during the end-of-life stage considering a worst-case scenario, leaching tests of Ag from the total immersion of Ag-WDs in Artificial Fresh Water (AFW), Artificial Marine Water (AMW) and soil:water extract were conducted. AFW was synthetized following (Organisation for Economic Co-operation and Development (OECD), 1992), while AMW was prepared following ASTM D1141-98:2021 ASTM International (2021). Soil: water extract was obtained by mixing LUFA 2.2 soil (LUFA Speyer, Germany) and ultrapure water in a proportion of 1:5 (w/v) with an orbital shaker for 5 min, at 250 rpm. Afterwards, the mixture was centrifuged for 20 min at 2000 rpm. The supernatant was collected and filtered through a 0.7 µm filter to avoid larger surface material. The pH of this medium resulted 5.4 ± 0.2 according to the analysis performed (Irizar et al., 2018).

Samples (i.e., 5 × 5 cm pieces of each Ag-WD) were analysed at days 1-3-7-14-21 and 28, where day 28 corresponds to the duration time of the sub-acute ecotoxicological tests.

For the analyses, an ICP-MS equipped with a seaFAST autosampler was employed using six points for the calibration curve in the range of 0.25–100 ppb adding the stock standard solution of Ag 1,000 ppm in a solution of HNO_3_ 2%. Duplicates were performed for each Ag-WD and presented as mean ± standard deviation of Ag content for each piece of Ag-WD (µgWD^−1^). Analysis was conducted in KED (kinetic energy discrimination) mode by using He as collision gas. Samples were automatically diluted 10 times and Y at 10 ppb was used as internal standard. The Limit of Detection (LoD) and the Limit of Quantification (LoQ) were automatically calculated by the software of the ICP-MS technique as the average of blanks +3 standard deviation (SD) and as the average of blanks +10 SD, obtaining a LoD of 0.09 ppb for AFW, 0.18 ppb for AMW and 0.49 ppb for soil:water extract, while LoQ were 0.24 ppb for AFW, 0.5 ppb for AMW and 1.3 for soil:water extract. As no reference certified materials are available on the market containing AgHEC NPs, accuracy has been assessed adding Ag and AgHEC NPs at the concentration of 100 mgL^−1^ in a solution of pure HNO_3_ (69%) and analysing samples after 6 h, obtaining a mean concentration of 95 ± 4%.

A colloidal characterization of Ag and AgHEC NPs in the different environmental media was also performed using Dynamic Light Scattering, Electrophoretic Light Scattering and Centrifugal Separation Analysis and described in SI2.

#### 3.2.4 Cost-effectiveness ratio

To estimate the effectiveness of the Ag-WDs as a function of costs, the Cost-Effectiveness ratio (CE%) between the total Ag content and the Ag released during immersion in synthetic sweat was calculated.

The total Ag content in Ag-WDs was determined by ICP-MS. Before ICP-MS analysis, one piece of each Ag-WD (5 × 5 cm) was weighted and immersed in 5 ml of HNO_3_ and after 6 h total dissolved Ag content (both particulate and ions Ag) was measured using ICP-MS equipped with a seaFAST autosampler. The method used was the same described in paragraph 3.1.3. LoD and LoQ values were automatically calculated by the ICP-MS software (as reported in paragraph 3.2.3) obtaining an LoD of 0.095 ppb and an LoQ of 0.26.

Considering leaching tests of Ag from Ag-WD immersed in synthetic sweat, as a controlled release of Ag needs to be guaranteed along the entire WD application, ICP-MS measurements of Ag after 1, 3 and 7 days of total immersion of the Ag-WD in synthetic sweat (simulating the worst-case scenario) were performed.

As demonstrated by Midander et al., 2016, the use of a comprehensive artificial sweat containing amino acids, vitamins, organic acids and carbohydrates for the evaluation of metal release do no significantly differ from the EN artificial sweat protocol, and for this reason, synthetic sweat was prepared according to the EN 1811:2011 protocol by mixing urea (0.1 wt%), sodium chloride (0.5 wt%) and lactic acid (0.1 wt%) in deionized water. A Scaltec SBA41 balance (readability: 0.001 g) was used for the weighing of the chemicals. The pH of the solution was adjusted with 1 M NaOH to reach the pH of 6.5 ± 0.05 (Hanna Instruments HI-5522-02 multiparameter meter).

One piece of each wound dressing (5 × 5 cm) was weighted, immersed in 50 ml of synthetic sweat and stored without agitation. Duplicates were performed for each Ag-WD and presented as mean ± standard deviation of Ag content for each piece of Ag-WD. When the selected time of immersion was reached, 0.25 ml from each sample were mixed with 2.25 ml of nitric acid (HNO_3_) 2% and analysed (indicated hereafter as µgWD^−1^).

For the analyses, ICP-MS measurements were obtained using the same methods described in paragraph 3.1.2 and adding the stock standard solution of Ag 1,000 ppm in a solution containing 200 ppm of NaCl, 300 ppm of CaCl_2_*2H_2_O and 100 MgCl_2_*6H_2_O, for simulating the salts contained in the synthetic sweat. Detection Limit automatically calculated by ICP-MS technique (as explained in paragraph 3.2.3) was 0.08 ppb and a LoQ of 0.22 for Ag element.

Measurements of the total Ag content and the Ag released during immersion in synthetic sweat was used to calculate the CE ratio using Equation 2:

$$CE_{t_{i}}=\frac{\left[\text{Ag}\;\text{tot}\right]}{\left[\text{Ag}\;{\text{released}}_{t_{i}}\right]\;}\times 100$$ (2)

Where $CE_{t_{i}}$ is the % ratio cost-effectiveness calculated at different time of immersion of the Ag-WDs, *[Ag tot]* is the total Ag concentration in wound dressings (µgWD^−1^) and $\left[Ag\;released_{t_{i}}\right]$ is the concentration of Ag released from the Ag-WDs (µgWD^−1^) at *i* time of immersion in synthetic sweat (e.g., after 1, 3 and 7 days).

### 3.3 Application of the SbD procedure: Experimental results

#### 3.3.1 Mechanical strengths of Ag-WDs

**FIGURE 2:**
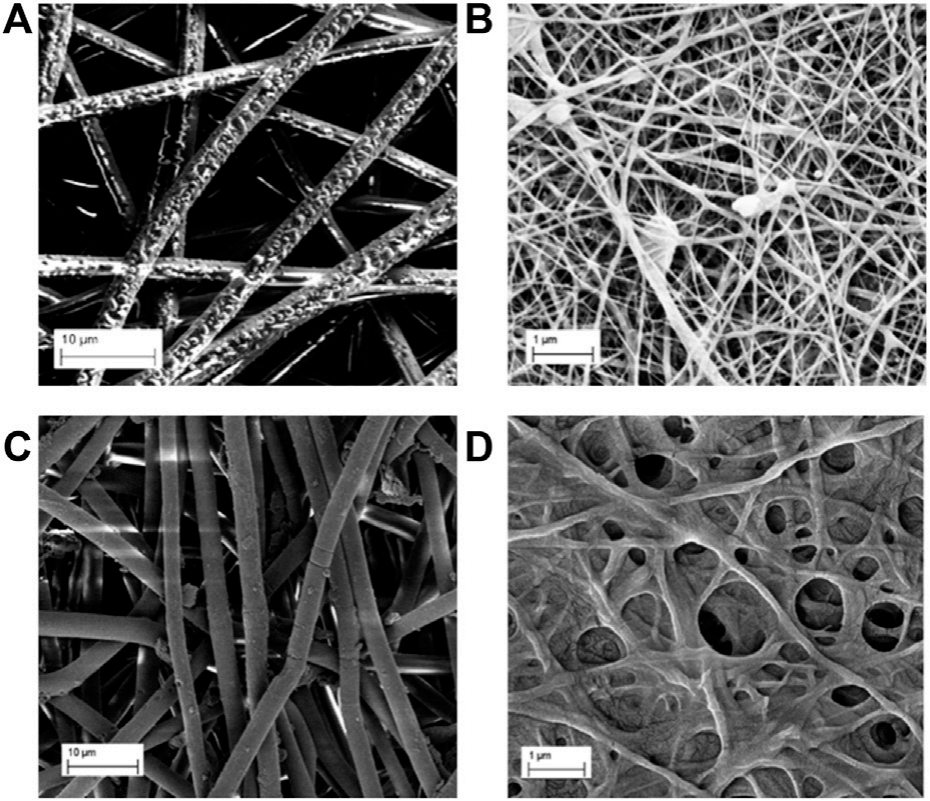
SEM images before immersion of **(A)** PLLA-Ag, **(B)** PLLA-AgHEC, and after 24 h of immersion in synthetic sweat of **(C)** PLLA-Ag and **(D)** PLLA-AgHEC.

**FIGURE 3:**
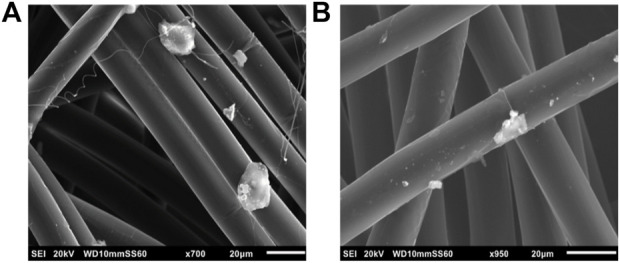
SEM images of PVA-Ag **(A)** before and **(B)** after 24 h of immersion in synthetic sweat.

**FIGURE 4:**
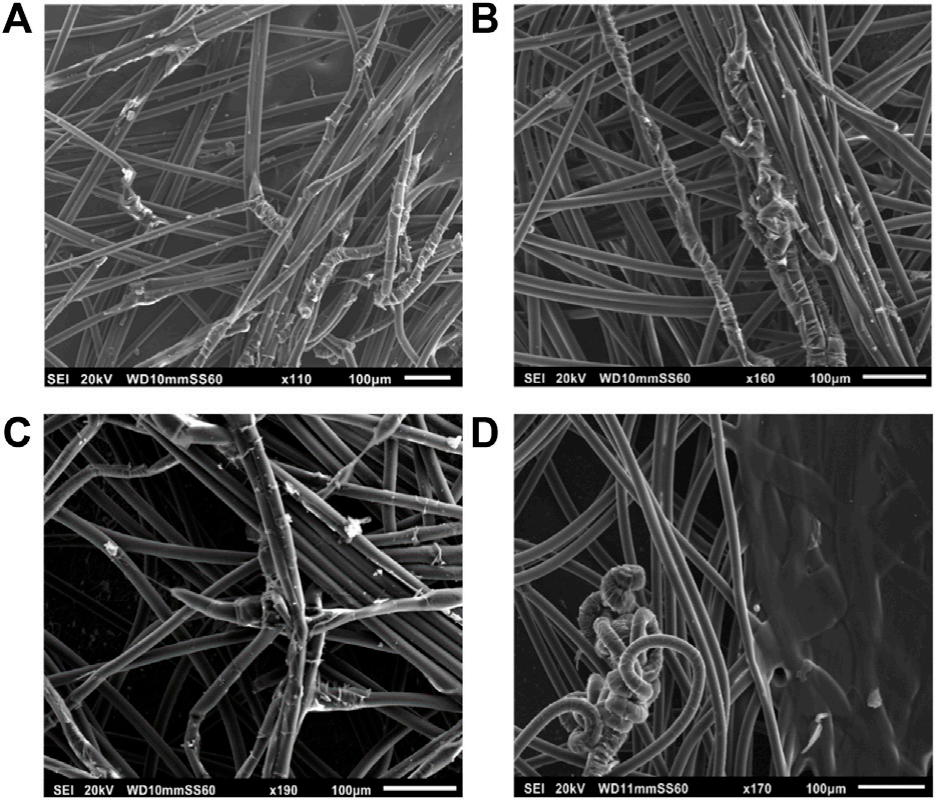
SEM images before immersion of **(A)** PVA-AgHEC.1 h, **(B)** PVA-AgHEC.2h, and after 24 h of immersion in synthetic sweat of **(C)** PVA-AgHEC.1 h and **(D)** PVA-AgHEC.2 h.

PLLA-Ag (Figure 2A) and PVA-Ag (Figure 3A) are composed of uniformly distributed electrospun fibers, while PLLA-AgHEC (Figure 2B), PVA-AgHEC.1 h (Figure 4A) and PVA-AgHEC.2 h (Figure 4B) showed an excess of polymer in small regions of the Ag-WD.

Fibres in PLLA-Ag sample are quite homogeneous in terms of size with a diameter of 2.5 ± 0.4 µm, and the observed morphology is preserved even after 24 h of immersion in synthetic sweat obtaining a value of 2.7 ± 0.3 µm (Figure 2C). In PLLA-AgHEC, PLLA fibres have a lower size than PLLA-Ag (i.e., diameter size of 70 ± 23 nm), and once they are immersed in synthetic sweat, the shape is not preserved, and the mechanical resistance affected (Figure 2D). Such detrimental effect on mechanical strength, probably due to the interaction of HEC with PLLA matrix, did not allow to calculate the diameter of fibres.

Considering PVA-Ag SbD alternative, shape and dimension of the fibres are maintained even after immersion in synthetic sweat (Figure 3B). Indeed, a diameter size of 15.4 ± 1.3 µm of this sample was obtained before immersion which is close to the value of 16.4 ± 2.1 µm after immersion. Methods and results obtained from EDX analysis of this sample are reported in SI3 and confirm the presence of Ag on the fibres before and after immersion in synthetic sweat.

Similarly, in PVA-AgHEC.1 h sample, shape and dimension of the fibres are preserved after immersion (Figure 4C), as indicated by the measured diameters (i.e., 16.4 ± 1.8 µm and 15.5 ± 1.8 µm before and after immersion, respectively). No significative differences are observed in PVA-AgHEC.2 h sample after immersion as well (Figure 4D), which before and after immersion confirmed similar fibres diameter values (i.e., 16.2 ± 1.5 µm and 16.3 ± 1.5 µm, respectively).

**FIGURE 5:**
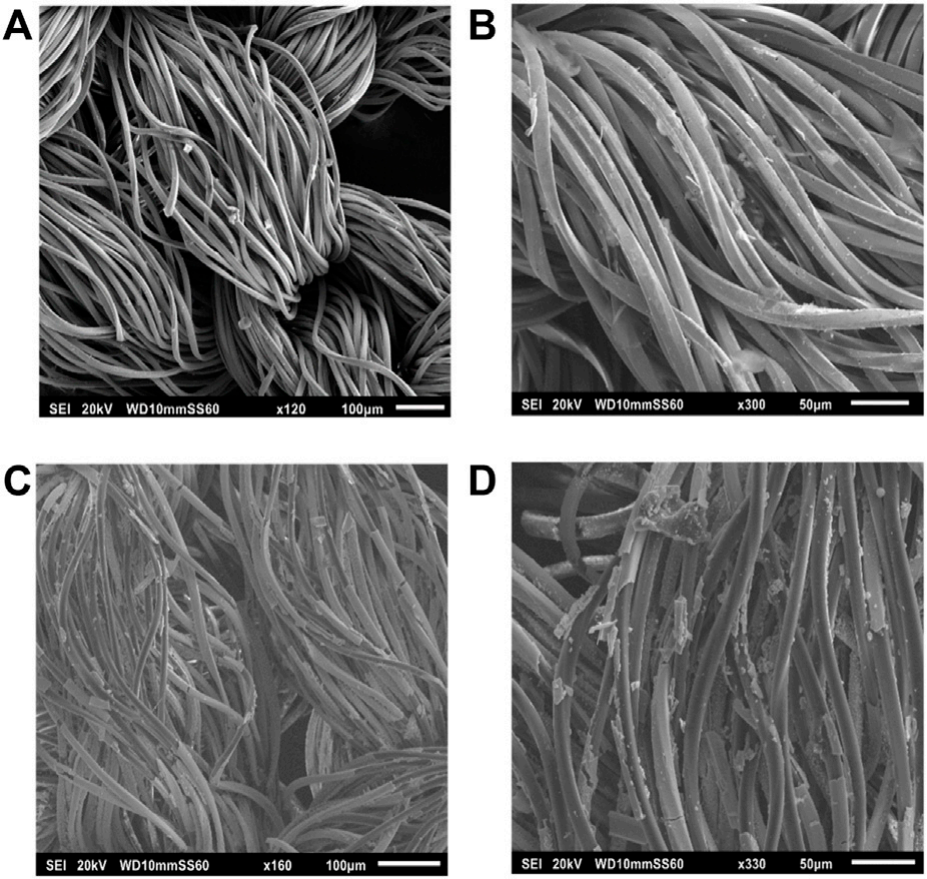
SEM images before immersion of **(A)** Acticoat Flex3, **(B)** Acticoat Flex7, and after 24 h of immersion in synthetic sweat of **(C)** Acticoat Flex3 and **(D)** Acticoat Flex7.

Considering commercial Ag-WDs, fibres of both Acticoat Flex 3 and Acticoat Flex 7 are homogeneous in size and shape (Figures 5A,B). However, after immersion in synthetic sweat (Figures 5C,D) a degradation of the fibres’ surface is observed. This degradation is probably related to a detachment of the external layer of the fibres, because of the interaction with the synthetic sweat. Comparing the fibres diameter before and after immersion in the simulated medium (where for WD after immersion, only diameters of fibres without the external layer were measured), a slightly increase in fibre size after immersion was observed for Acticoat Flex 3 (i.e., from 6.8 ± 1.9 µm to 7.7 ± 0.8 µm before and after immersion), while for Acticoat Flex7, a slightly decrease in size after immersion in sweat was obtained (i.e., from 9.8 ± 1.6 µm to 7.8 ± 1.3 µm).

To conclude, because of the low mechanical strength of fibres after immersion in synthetic sweat, PLLA-AgHEC was not further investigated as SbD alternative. On the other hand, PLLA-Ag and PVA-based samples pointed out a mechanical resistance under simulated using condition in line with the commercial products.

#### 3.3.2 Antimicrobial tests of Ag-WDs against *E. coli*

The antibacterial performance measured on Ag and AgHEC suspensions showed a boosted biocidal action for AgHEC. In fact, a total bacterial depletion of 100% was obtained at high concentration (130 mg L^−1^) and also after dilution. An antibacterial reduction of 94% was indeed observed for HEC polymer without NPs. For Ag Sigma NPs suspension, the antimicrobial efficiency was almost total (99%) at 130 mg L^−1^ but decreased to 64% at the Ag concentration of 13 mg L^−1^.

To deeper investigate the antibacterial functionality of Ag-based compounds once embedded into the fibres, the antimicrobial activity was determined on Ag-WD surfaces as well.

Indeed, the best antibacterial effect among the four WDs against Gram-negative bacteria *Escherichia coli* reduction are shown by both samples based on PVA-AgHEC fibers (i.e., 100% antibacterial efficacy) followed by PLLA-Ag and PVA-Ag (Supplementary Table SI4). As expected, the antimicrobial efficacy of PVA-AgHEC is induced by AgHEC, as PVA and PLLA polymers did not show any antibacterial activity (i.e., 27 and 69% for PVA and PLLA respectively).

#### 3.3.3 Leaching tests of Ag from Ag-WDs immersed in environmental media

Leaching tests of Ag from Ag-WDs at different time of immersion were performed in AFW, in AMW, and in soil:water extract and results are reported in Supplementary Table SI4.

Considering Ag released from SbD Ag-WDs alternatives immersed in AFW, the lowest values of Ag were observed for PLLA-Ag WDs (range between 1 and 3 µgWD^−1^), while the amount of Ag released from PVA-Ag WDs reached the highest value at day 28 (around 25 µgWD^−1^). As far as the SbD WDs containing AgHEC NPs are concerned, the amount of Ag determined from both PVA-AgHEC.1 h and PVA-AgHEC.2 h was almost constant, showing negligible differences among the concentrations of Ag released at the different immersion time selected. While the release of Ag from the SbD WDs resulted in the range between 1 and 25 µgWD^−1^, the release of Ag from the commercial Ag-WDs resulted much higher, with concentrations ranging from 230 to 400 µg. In addition, for both commercial WDs, the release of Ag slightly increased from day 1 and day 28 and the concentrations of Ag released from Acticoat Flex 7 were always higher than those measured for the Acticoat Flex 3 at each time of immersion.

According to the obtained leaching tests of Ag from Ag-WDs immersed in AFW, the SbD alternatives can be ranked as follows: PLLA-Ag > PVA-AgHEC.1 h > PVA-AgHEC.2 h > PVA-Ag.

Considering leaching tests of Ag from Ag-WDs in AMW, PVA-AgHEC.1 h WDs showed the lowest Ag release values in almost all the time of immersion (between 7 and 26 µgWD^−1^), while Ag released from PVA-AgHEC.2 h WDs reached the highest values at each time of immersion (range between 25 and 82 µgWD^−1^). Comparing WDs containing uncoated Ag NPs, the leaching of Ag from PLLA-Ag WDs was lower at day 1 and 3 than that observed for PVA-Ag WDs, while from day 7 up to day 28 an opposite behaviour was observed. These results can be ascribed to the different Ag-WDs fibre structure, which is related to the two different polymers used in the Ag-WDs. As already observed for AFW, the Ag released from the commercial Ag-WDs immersed in AMW (180-250 µgWD^−1^) resulted always higher than those released from the SbD alternatives (4-58 µgWD^−1^), and the concentrations determined from the immersion of Acticoat Flex 7 in AMW were always higher than those obtained from Acticoat Flex 3 at each time of immersion.

According to the obtained leaching tests of Ag from Ag-WDs immersed in AMW, the SbD alternatives can be ranked as follows: PVA-AgHEC.1 h > PVA-Ag > PLLA-Ag > PVA-AgHEC.2 h.

Leaching tests of Ag from SbD WDs immersed in soil:water extract revealed that the lowest concentration of Ag was released from PLLA-Ag at each time of immersion (range from 3 to 5 µgWD^−1^), while the highest amount of Ag was released from PVA-AgHEC.2 h WDs (range 25-34 µgWD^−1^). Similar results were observed for PVA-Ag WDs and PVA-AgHEC.1 h WDs, showing releases ranging from 12 to 18 µgWD^−1^ and from 8 to 12 µgWD^−1^ respectively.

Similarly to what observed in AFW and AMW, the leaching of Ag from the commercial Ag-WDs immersed in soil:water extract were much higher than those obtained from the SbD alternatives. Acticoat Flex 7 for example released 10 mgWD^−1^ of Ag after 28 days of immersion in the soil:water extract. As reported from the literature, the pH of the medium, as well as the presence of dissolved organic matter, can influence the stability of Ag NPs (Reidy et al., 2013), leading to higher dissolution of Ag NPs at lower value of pH (Liu & Hurt, 2010; Liu et al., 2012). Accordingly, the high release values of Ag from the two commercial Ag-WDs (3-10 mgWD^−1^) can be related to the acidic pH of the soil:water extract.

From the obtained results, the SbD alternatives can be ranked as follows: PLLA-Ag > PVA-AgHEC.1 h > PVA-Ag > PVA-AgHEC.2 h.

#### 3.3.4 Cost-effectiveness ratio

Values obtained from ICP-MS measurements of the total Ag in Ag-WDs and Ag released from Ag-WDs immersed in synthetic sweat are reported in Supplementary Table SI4, where % of Cost Effectiveness index (CE) is calculated for each Ag-WDs at day 1, 3 and 7.

The amount of Ag contained in each piece of Ag-WDs (5 × 5 cm) was determined by ICP-MS analysis, and the results obtained are reported in Supplementary Table SI4 as µgWD^−1^. As can be observed from the table, the total amount of Ag contained in the commercial Ag-WDs is higher than those determined in the SbD alternatives selected (30 mgWD^−1^ of Ag for the commercial Ag-WDs vs amounts ranging from 40 µgWD^−1^ to 2 mgWD^−1^ for the SbD alternatives). Moreover, as expected, Acticoat Flex 7 showed a higher amount of Ag than Acticoat Flex 3, since its controlled release of Ag up to 7 days. Considering SbD alternatives, PLLA-Ag showed the highest Ag content (around 2 mgWD^-1^) compared to the other Ag-WDs (between 40 and 125 µgWD^-1^), followed by PVA-Ag > PVA-AgHEC.2 h > PVA-AgHEC.1 h.

The amount of Ag released from the selected Ag-WDs in the synthetic sweat was determined by ICP-MS at different immersion time. The overall results are reported in Supplementary Table SI4.

As it can be observed from Supplementary Table SI4, the concentrations of Ag released from the six samples analysed at day 1 and 3 in the synthetic sweat, ranged from 1 to 80 µgWD^−1^. The highest content of Ag was released from Acticoat Flex 3 and 7 (between 60 and 80 µgWD^−1^). These results were expected since these materials present a higher initial amount of Ag than the other SbD alternatives (see chapter 3.1.2). On the other side, the lowest release of Ag was detected for PLLA-Ag WDs (ranging from 1-10 µgWD^−1^), while the amount of Ag released from PVA-Ag WDs ranged from 8 to 30 µgWD^−1^.

Measurements obtained after 1, 3 and 7 days of immersion were always higher than those observed for the PLLA-Ag WDs, despite the initial Ag amount of PLLA-Ag which is one order of magnitude higher than PVA-Ag (see chapter 3.1.2). Considering AgHEC WDs, the amount of Ag released from PVA-AgHEC.1 h WDs (between 10 and 18 µgWD^−1^) was twice less than the Ag released form PVA-AgHEC.2 h WDs (range between 25 and 50 µgWD^−1^). These results can be ascribed to the different time used in the electrospinning process (1 vs 2 h), which is also related to the initial Ag contained in the Ag-WDs.

Considering SbD alternatives, PLLA-Ag showed the lowest %CE value (i.e., 0.4%), indicating that the high quantity of Ag present on the PLLA fibres is almost not released. On the contrary, % CE of both PVA-AgHEC.1 h and PVA. AgHEC.2 h WDs were close to 26% at day 1, at 30% at day 3 and 45% at day 7, suggesting the high effectiveness of these Ag-WDs. For commercial Ag-WDs, the %CE was always very low (from 0.1 to 0.4%) suggesting that despite the high quantity of Ag added to the polymer, the Ag is released only in very small quantity.

According to the obtained results, SbD alternatives can be ranked as follows: PVA-AgHEC.1 h > PVA-AgHEC.2 h > PVA-Ag > PLLA-Ag.

### 3.4 SbD evaluation: Final ranking

According to the experimental results, the ranking of Ag-WDs alternatives for each SbD criterion is reported in Table 2, where the three criteria have equal weight in the final assessment and the SbD alternatives are coloured from dark green (best option) to light yellow. This summary facilitates the identification of the most promising option based on the selected criteria, that is the option with the highest number of dark green cells: the top-ranked option is PVA-AgHEC.1 h. Indeed, this Ag-WD turned out to be the best alternative as for antimicrobial efficacy, cost-effectiveness and leaching of Ag in AFW criteria and ranks very well also in leaching of Ag in AMW and in soil: water extract criteria.

**TABLE 2:** Ranking of the Ag-WD alternatives according to the investigated SbD evaluation criteria. The three criteria have equal weight in the final assessment.

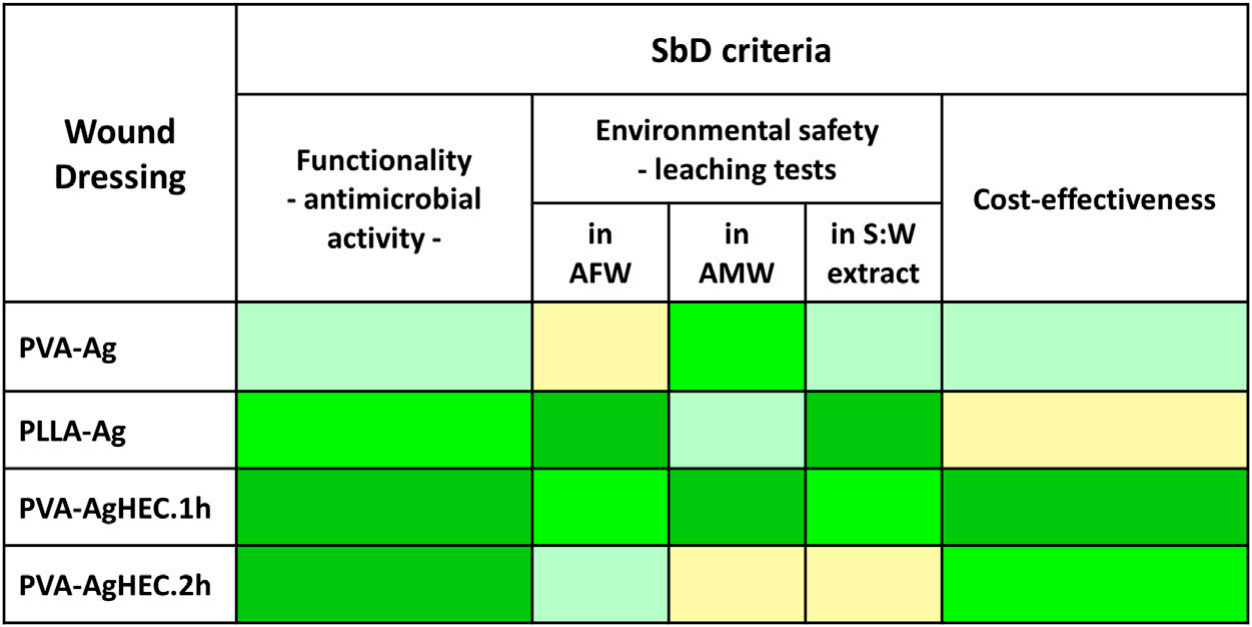

AFW: artificial fresh water, AMW: artificial marine water, S:W extract: soil:water extract. Colours-dark green: the best alternative, light green: medium alternatives, yellow: the worst alternative.

## 4 Conclusion

This manuscript proposes a novel methodology to prioritise SbD alternatives of Ag-WDs based on criteria pertaining to environmental safety, functionality, and economic viability. This approach was inspired by the SbD approaches found in literature and was adapted based on the Risk Management Framework developed in Giubilato et al., 2020 for nano-biomaterials used in medical devices and Advanced Therapy Medicinal Products.

The proposed methodology was applied to five experimental Ag-WDs, and to two commercial wound dressings for a comparison with products already on the market.

It involves two main steps: material design and SbD evaluation. In the first step, SbD objectives were identified, which are 1) maximisation of the antimicrobial activity of the Ag-WD, 2) reduction of possible Ag released into the environment, 3) optimization of the cost-effectiveness of the Ag-WD. Then, for each of these objectives, ad-hoc tests and assessments were selected and performed to generate relevant information on criteria to assess the functionality, safety, and economic viability of the investigated wound dressings. The identified criteria and tests were considered necessary and sufficient for the specific product and the selected level of analysis (i.e., screening assessment). The application of the proposed methodology to different wound dressings, characterized by e.g., different mode of action and duration of application, would require a revision of both SbD criteria and tests. Moreover, for a different level of analysis (i.e., advanced assessment once the production is scaled up) the application of more complex tools is advisable (e.g., Life Cycle Assessment, Risk Assessment).

The SbD procedure, intended for the early steps of wound dressing development, allowed to discard a nano-enabled wound dressing before further resources were invested and to support the selection of the best SbD alternative, according to the following criteria: mechanical strength of the Ag-WDs, their antibacterial effect, release of Ag from WDs immersed in environmental media, and cost-effectiveness. The Ag-WDs were ranked based on the criteria, which led to identifying the PVA-AgHEC.1 h as the safest alternative, which is, at the same time, sufficiently functional and economically viable.

However, while the presented procedure supported a preliminary analysis for the selection of the most promising alternatives, more in depth analysis of their human health and environmental safety need to be performed on the alternative candidates for further development. For example, as the antimicrobial effect of Ag is exerted by Ag^+^, Ag NPs and Reactive Oxygen Species (ROS) (Nešporová et al., 2020), an in depth analysis on the formation of ROS, stress response or cytotoxicity should be implemented to better scrutinize the safety assessment of Ag-WDs. Moreover, as Ag^+^/NPs can enter the circulatory system through the application of the wound dressings on the ulcered wounds, the behaviour of Ag in the synthetic blood can be an additional aspect to investigate in further analysis.

Additional environmental criteria could be also included in further SbD assessment to improve the understanding of environmental implications of new nano-enabled products. For instance, further investigations could be performed to study not only the behaviour of Ag NPs and Ag^+^ under different environmental conditions (e.g., sunlight, oxygen-rich water, sulphide-rich water) (Zhang et al., 2018) but also considering short- and long-term ecotoxicological effects about the investigated Ag NPs on aquatic and terrestrial organisms.

This multicriterial SbD methodology can be modified in future works also considering other categories of nano-enabled products used in medicine (e.g., antimicrobial sprays, tooth filling paste) by modifying the SbD criteria according to their specific characteristics, their intended use and expected processes occurring during the end-of-life stage, and adding criteria pertaining to the sustainability (including circularity) of the products in order to support Safe and Sustainable by Design decision making.

## Data Availability

The raw data supporting the conclusion of this article will be made available by the authors, without undue reservation.
