# Supplementary material for: Identification of the safe(r) by design alternatives for nanosilver-enabled wound dressings
Source: Front Bioeng Biotechnol. 2022 Oct 11;10:987650. doi: 10.3389/fbioe.2022.987650 (PMC9614711; doi:10.3389/fbioe.2022.987650)
Supplement: Supplementary file 1 [file DataSheet1.PDF]

Supplementary Material

Appendix SI1. Synthesis and characterization of the test samples

According to ISO 10993-22:2017 on the "Biological evaluation of medical devices. Part 22: Guidance on nanomaterials", the five Ag-WDs alternatives considered in this study can be classified as medical devices with an intentional release of NPs. These Ag-WDs were designed to release Ag up to 7 days in acute and chronic wounds.

The investigated Ag-WDs were developed using the electrospinning process, a technique capable of producing fibres from polymers with diameters in the nano- to micrometer range containing NPs (Rujitanaroj et al., 2008). Such process ensures the development of uniform and stable fibrous scaffolds (An et al. 2013, Alberti et al. 2020) and fibres that act as an effective barrier for damaged skin to prevent pathogens (Augustine et al., 2018). Because of their biocompatibility, biodegradability and low cost (Augustine et al., 2018; Gökmeşe et al., 2013; Thamarai Selvi et al., 2018), polyvinyl alcohol (PVA) and poly-L-lactide (PLLA) has been used as matrix for the Ag-WDs (Ambekar & Kandasubramanian, 2019), while the antimicrobial efficacy was exerted by two types of Ag NPs: uncoated Ag and Ag coated with hydroxyethyl cellulose (HEC).

Since the concentration range of Ag NPs needed to reach an antimicrobial efficacy is not reported in the literature (White & Cutting, 2006), the optimal amount of Ag NPs to be included in the Ag-WDs cannot be established before performing the antimicrobial tests. Therefore, the five SbD alternatives differ not only in the type of Ag NPs but also in the total Ag content.

The first type of Ag NPs considered in this study are commercial NPs from Sigma Aldrich (referred to as Ag Sigma NPs). These NPs are uncoated and with a size distribution ranging approx. from 10 to 150 nm, as measured with Transmission Electron Microscopy (TEM) (results are reported in the following paragraphs).

The second type of NPs are Ag NPs coated with HEC synthetized following a patented procedure (Costa & Blosi, 2016). The coupling of Ag NPs with a positively charged polymer provided a key synergistic effect in antimicrobial activity with enhanced performances against pathogenic strains compared to commercial Ag NPs. Furthermore, the HEC coating enhances the Ag interaction with polymeric/organic formulations and contributes to improve the Ag biocompatibility. The synthesis of AgHEC NPs is an eco-friendly process, entirely carried out at room temperature, by using safe reagents and water as solvent. The synthesis enables the achievement of high concentrated and stable suspensions (0.1-0.5% wt) with a size of 9 ± 1 nm (reported in the following paragraph).

Considering the polymer matrix, commercial PVA with an average molecular weight of 130000 g mol^{-1} was purchased from Sigma-Aldrich (Italy). PVA powder was dissolved as received in water at 90°C, under mild stirring, until the solution appeared transparent (about 2 h). PVA solution was slightly cooled down at ambient temperature (about 20 °C) under stirring. Then, 5 mg of Ag Sigma NPs powder were added to 10 mL of PVA solution. PVA-Ag solution were electrospun using electrospinning equipment consisting of a high-voltage generator (Spellman SL300P, USA) electrically connected to a 27G metal tip (Butterfly infusion set by Hospira, UK), a metering pump (KDS 200 from KD Scientific) feeding the solution to the metal tip (0.4 mm internal diameter) and a flat metal collector (50 x 50 cm) electrically grounded (for Ag Sigma). A 23 gsm polypropilene spunbonded non-woven with an average fibre size of 16 ± 4 μm (supplied by Soft NW, Italy) was cut in squares with the same size of the collector and stuck on it as a substrate suitable for handling the nanofiber layers. The PVA-

Ag solutions were processed at voltages of +30 kV at the tip with a working distance from the tip to the collector of 20 cm and a flow rate of 0.02 $mLmin^{-1}$. The ambient conditions were 21 ± 2 °C temperature and 35 ± 2 % relative humidity. Electrospun nanofibers were collected on the non-woven and the PVA-Ag WD was obtained.

Colloidal AgHEC NPs dispersion was added to the PVA solution at a volume ratio 1:1. The final hybrid PVA-AgHEC solutions were kept under stirring for at least 2 h in order to ensure complete mixing before electrospinning. The PVA-AgHEC solutions were processed at voltages of +30 kV at the tip and −5 kV at the collector with a working distance from the tip to the collector of 20 cm and a flow rate of 0.02 $mLmin^{-1}$. The ambient conditions were 22 ± 2 °C temperature and 35 ± 5 % relative humidity. Electrospun nanofibers were collected on the non-woven. Each deposition lasted 1 h and 2 h, developing wound dressing called PVA-AgHEC.1h and PVA-AgHEC.2h respectively and both containing 0.7% wt of Ag calculated on dry fibres.

Ag Sigma and AgHEC NPs were then incorporated into electrospun PLLA fibers by the Electrospinning Company (UK) leading to PLLA-Ag WD and PLLA-AgHEC WD, respectively. Ag concentration calculated on dry fibres corresponds to 5%wt for PLLA-Ag WD and 4%wt for PLLA-AgHEC WD.

Two commercial wound dressings containing Ag NPs, namely Acticoat Flex3 and Acticoat Flex7, were selected, analysed, and, considering their ability to constantly release Ag during their application period (i.e., 3 and 7 days respectively), were compared with the different SbD alternatives.

Transmission Electron Microscopy analysis of Ag and AgHEC NPs

Transmission Electron Microscopy analysis (TEM) was performed by means of a FEI TECNAI F20 instrument microscope operating at 200 keV. Ag suspensions were drop-casted on a perforated carbon film supported by a gold grid and the specimen dried at 40 °C. To gather information about particles morphology, TEM images were collected in a phase contrast mode.

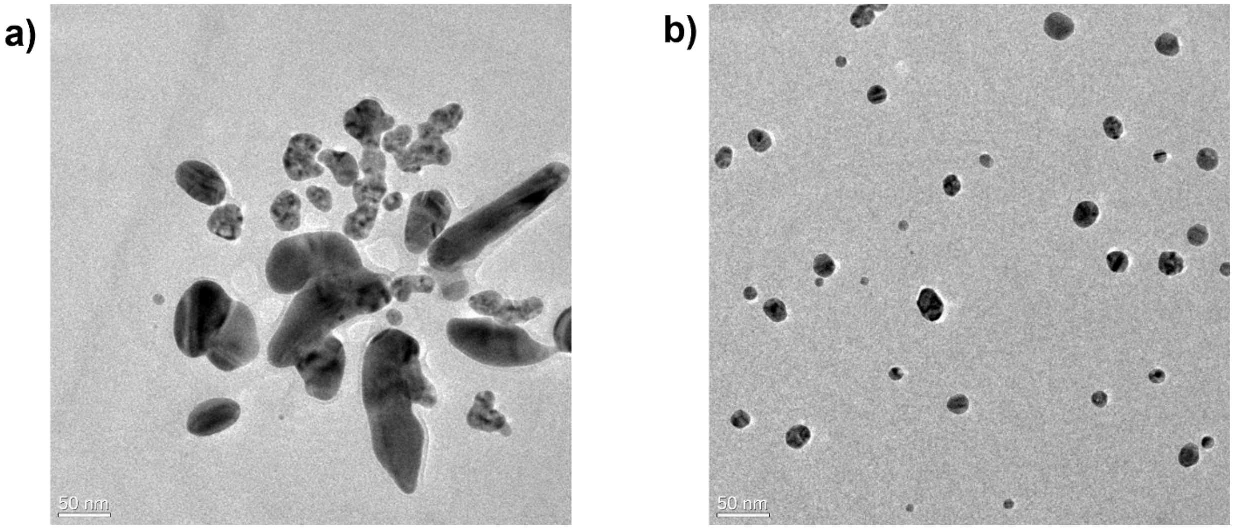

Figure 1.TEM images collected for a) Ag Sigma and b) AgHEC NPs

The collected TEM images showed for Ag Sigma crystalline particles with irregular morphology and size ranging from 10 to 150 nm (Figure 1a). AgHEC images highlighted spherical regular nanoparticles morphology with diameter ranging from 3 to 20 nm with a mean diameter of 9±1 nm (Figure 1b) calculated on more than 150 particles by imaging analysis using Image J software.

Appendix SI2. Colloidal characterization of NPs in environmental media

The concentrations of Ag and AgHEC NPs used for the analyses (i.e., 1-10-100 mgL^{-1} of Ag NPs) were selected in order to reach the lowest concentrations of NPs detectable from Dynamic Light Scattering (DLS), Electrophoretic Light Scattering (ELS) and Centrifugal Separation Analysis (CSA) techniques and to be as close as possible to the concentrations of Ag released from the pieces of Ag-WDs immersed in the three environmental media.

Ag and AgHEC were weighted using a Cubis Sartorius balance and dispersed in AFW, AMW, soil:water extract, and in ultrapure water (to investigate the behaviour of Ag NPs in the absence of salts). Ultrahigh-pure water (UPW, minimum resistivity: 18.2 MΩ·cm) was produced by a MilliQ water purifier system (Millipore, Bedford, MA, USA).

As the use of sonication in NPs dispersions (required by dispersion protocol such as NanoGenotox) can increase particle dissolution and change surface properties of metal NPs (Pradhan et al., 2016), Ag and AgHEC NPs were added in the medium and then manually shaken.

DLS measurements were performed by means of the multi-angle Nicomp ZLS Z3000 (Particle Sizing System, Port Richey, FL, USA) to determine the particle size distribution of the NPs. Hydrodynamic diameter (d_H) was measured with an optical fiber set at 90° scattering angle (W=25 mW and λ=639 nm) at room temperature. Refraction index of 1.333 and viscosity value of 0.993 cP were used as formulations are dispersed in water-based media.

ELS measurements were obtained using a Zetasizer Nano instrument ZSP, ZEN5600, Malvern UK. A 5V electric field was applied and a zeta potential (ζ-pot) was determined from the mean phase shift with respect to time. The Smoluchowski equation was applied to convert the electrophoretic mobility to the zeta potential.

Both d_H and ζ-pot values were obtained according to three independent measurements, with each measurement consisting of three individual readings and presented as mean ± standard deviation.

CSA was employed to assess dispersion stability of NPs in terms of sedimentation velocity (V-sed) by using the Multiwavelength Dispersion Analyzer LUMiSizer® 651. This technique allows to compare different colloidal dispersions and to establish a stability ranking under specific experimental conditions. The temperature was set at 25°C throughout the time span of analysis. Sedimentation velocity values was achieved at 4000 Rotation Per Minute (RPM), which corresponds to a Relative Centrifugal Force (RCF) of 2146 at 120 mm far from the rotor of the centrifuge. Sedimentation velocity data can be calculated from the transmittance values obtained setting the wavelength of the transmitted light at 470 nm and collecting the transmittance (%) over time at three different positions (115, 120 and 125 mm far from the rotor) over the length of the cuvette. The runtime of each analysis was chosen according to the lowest time needed to reach the plateau, i.e., the maximum transmittance values, indicating the complete sedimentation of NPs. V-sed values are presented as mean ± standard deviation of three independent measurements.

The overall data of sedimentation velocity, V-sed, hydrodynamic diameter, d_H, and zeta potential, ζ-pot, are reported in Table 1.

Table 1. Sedimentation velocity (V-sed), hydrodynamic diameter (d_H), zeta potential (ζ-pot) of Ag and AgHEC NPs in ultrapure water, AFW, soil:water extract (S:W).

Sample name	NPs concentration (mg/L)	Medium	CSA		DLS		ELS	
			V-sed. (μm/s)	SD	d_H (nm)	SD	ζ-pot. (mV)	SD
Ag Sigma	100	AFW	Under LoD		421	305	-12	2
	10	AFW	Under LoD		Under LoD		Under LoD	
	1	AFW	Under LoD		Under LoD		Under LoD	
	100	AMW	Under LoD		510	87	-12	1
	10	AMW	Under LoD		Under LoD		Under LoD	
	1	AMW	Under LoD		Under LoD		Under LoD	
	100	S:W	0.66	0.2	1147	147	-10	1
	10	S:W	Under LoD		1493	208	-18.0	0.5
	1	S:W	Under LoD		1305	179	-4.0	0.6
	100	milliQ	Under LoD		796	134	-12	2
	10	milliQ	Under LoD		Under LoD		Under LoD	
	1	milliQ	Under LoD		Under LoD		Under LoD	
AgHEC	100	AFW	Under LoD		490	71	12.0	0.6
	10	AFW	Under LoD		Under LoD		Under LoD	
	1	AFW	Under LoD		Under LoD		Under LoD	
	100	AMW	Under LoD		730	120	7	2
	10	AMW	Under LoD		Under LoD		Under LoD	

	1	AMW	Under LoD		Under LoD		Under LoD	
	100	S:W	0.13	0.007	2209	329	12.1	0.6
	10	S:W	0.17	0.006	3024	464	-11.0	1
	1	S:W	0.14	0.02	1407	203	-10	1
	100	milliQ	Under LoD		970	152	6	1
	10	milliQ	Under LoD		Under LoD		Under LoD	
	1	milliQ	Under LoD		Under LoD		Under LoD	
**S:W**	S:W	S:W	0.26	0.02	1333	191	-11	1

AFW

Ag NPs dispersed in AFW were detected by DLS and ELS techniques only at the highest concentration tested (100 mgL^{-1}) with a d_H of around 420 nm and a negative ζ-pot value (-11.6 mV), the other tested concentrations were too low to be detected by these techniques. AgHEC could also be detected only by DLS and ELS at the highest concentration tested, showing a d_H similar to Ag NPs (of around 490 nm) but with a positive ζ-pot value (+12 mV), related to the presence of HEC polymer.

Comparing the behaviour of NPs in AFW and ultrapure water, d_H values of Ag Sigma NPs in AFW were observed smaller than in ultrapure water (around 796 nm) but with similar ζ-pot values (about -12 mV). Also for AgHEC NPs, smaller d_H values wer detected in AFW compared to d_H values in ultrapure water (around 970 nm), while the ζ-pot value increased from +6 to +12 mV moving from ultrapure to AFW.

As far as CSA results, transmission profiles of Ag NPs in AFW indicate that settling is occurring (Figure 2b). However, V-sed could not be measured for this sample since the initial transmission value was too high (at around 80%) – values would not be accurate. Moreover, transmission profiles of Ag Sigma NPs in AFW were similar to those detected for Ag Sigma in ultrapure water (Figure 2a).

In the case of AgHEC NPs in AFW (Figure 2d), the transmission profiles showed by the CSA were similar to those of AgHEC in ultrapure water (Figure 2c) and do not permit to calculate a V-sed also for AgHEC even at 100 mgL^{-1}.

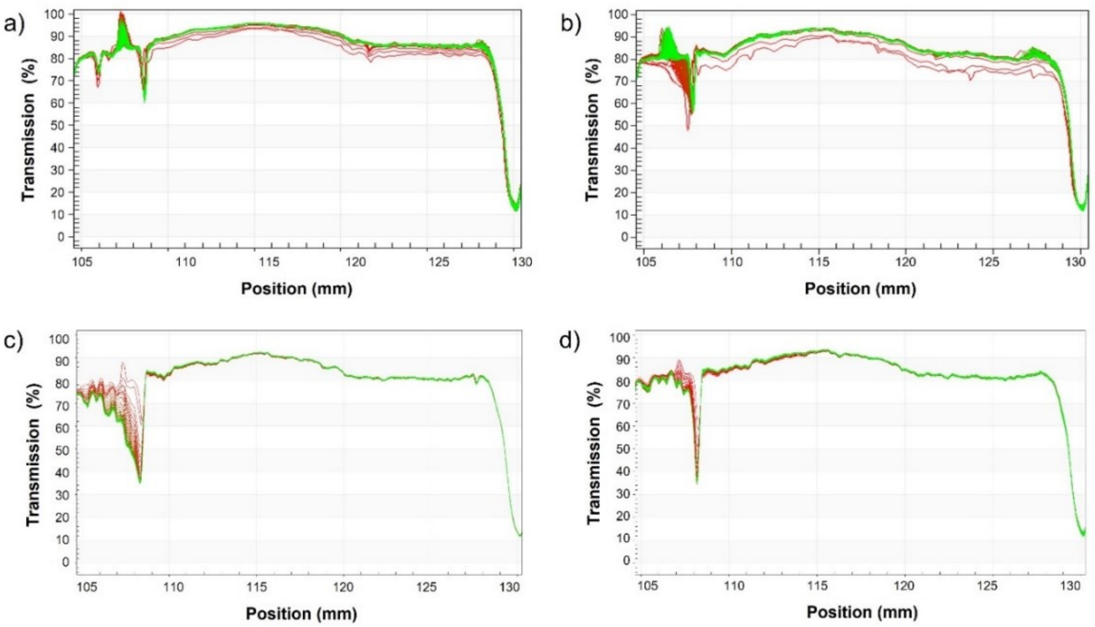

Figure 2. Transmittance profiles at 100mgL^{-1} of a) Ag NPs in ultrapure water, b) Ag NPs in AFW, c) AgHEC NPs in ultrapure water, d) AgHEC NPs in AFW.

<u>AMW</u>

Because of the limit of detection of DLS and ELS techniques, d_H and ζ-pot values of Ag NPs in AMW were measured only at the highest concentration tested (100 mg·L^{-1}) showing a d_H of around 510 nm and a negative ζ-pot value (-11.8 mV). AgHEC could also be detected only at the highest concentration tested, obtaining a d_H of around 730 nm and a positive ζ-pot value (+7.2 mV) because of the presence of positive charged HEC polymer. d_H values were slightly smaller than those obtaining at the same concentration in ultrapure water (i.e., 790 and 970 nm for Ag and AgHEC respectively), while ζ-pot values of both the dispersions at 100 mg·L^{-1} in AMW were similar to those measured in ultrapure water (i.e., -12.6 for Ag and 6.3 for AgHEC).

As far as CSA results, transmission profiles of Ag and AgHEC NPs in AMW at 100 mg·L^{-1} (Figure 3a and 3b respectively) were similar to transmission profiles of ultrapure water (Figure 3a and 3c) and V-sed values cannot be measured for both samples. For Ag and AgHEC NPs dispersions at 1 and 10 mg·L^{-1}, no measurements were obtained as the concentrations were too low to be detected by DLS, ELS and CSA techniques.

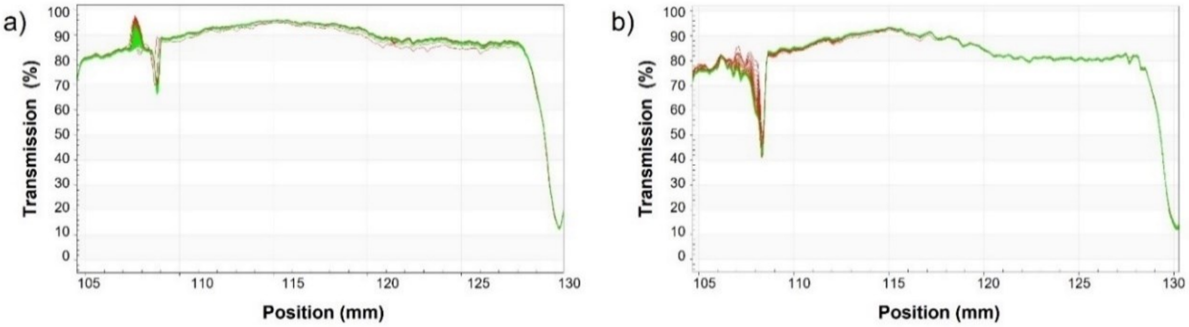

Figure 3. Transmission profiles at 100 mgL^{-1} of a) Ag NPs and b) AgHEC NPs in AMW.

Soil:water extract

Soil:water extract medium showed the presence of dispersed particles (according to the preparation protocol it can contain particles up to 50 µm). Therefore, the medium was characterized by DLS, ELS and CSA techniques without adding NPs. According to these techniques, particles with a hydrodynamic size of around 1300 nm, a negative ζ-pot value of around -7.0 mV and a V-sed value of 0.26 $\mu m \cdot s^{-1}$ were observed (Figure 4a). These suspended particles can interact with NPs (e.g., heteroaggregation processes) influencing the measurements, and, depending on NPs concentration, soil particles can completely overlap the signals of the investigated NPs.

As it can be observed from Table 1, d_H values measured for both Ag NPs at 10 and 1 $mg \cdot L^{-1}$ correspond to those of soil:water extract medium alone. A V-sed value of 0.66 $\mu m \cdot s^{-1}$ was obtained only at the highest concentration tested (Figure 4b).

Once AgHEC NPs are dispersed in soil:water extract, the HEC polymer probably interacts with particles in the soil medium. Indeed, transmittance profiles obtained at 100 $mg \cdot L^{-1}$(Figure 4c), at 10 $mg \cdot L^{-1}$ (Figure 4d), at 1 $mg \cdot L^{-1}$ (Figure 4e) showed a decrease of the sedimentation process of the sample, obtaining sedimentation velocity values of 0.13 $\mu m \cdot s^{-1}$ at 100 $mg \cdot L^{-1}$, 0.17 $\mu m \cdot s^{-1}$ at 10 $mg \cdot L^{-1}$ and 0.14 $\mu m \cdot s^{-1}$ at 1 $mg \cdot L^{-1}$. At these concentrations, hydrodynamic diameter values were higher than AgHEC in ultrapure water which can be detected only for 100 mgL^{-1}. Considering zeta potential values, the positive value of 12±0.6 mV at 100 $mg \cdot L^{-1}$, the negative values of -11mV at 10 $mg \cdot L^{-1}$and -9 mV at 1 $mg \cdot L^{-1}$ suggest that HEC was revealed at the highest tested concentration as HEC is a positively charged polymer. Indeed, a positive zeta potential value was obtained also in AFW (12±0.6 mV) and in ultrapure water (6.3±1 mV) at the same AgHEC concentration of 100 $mg \cdot L^{-1}$.

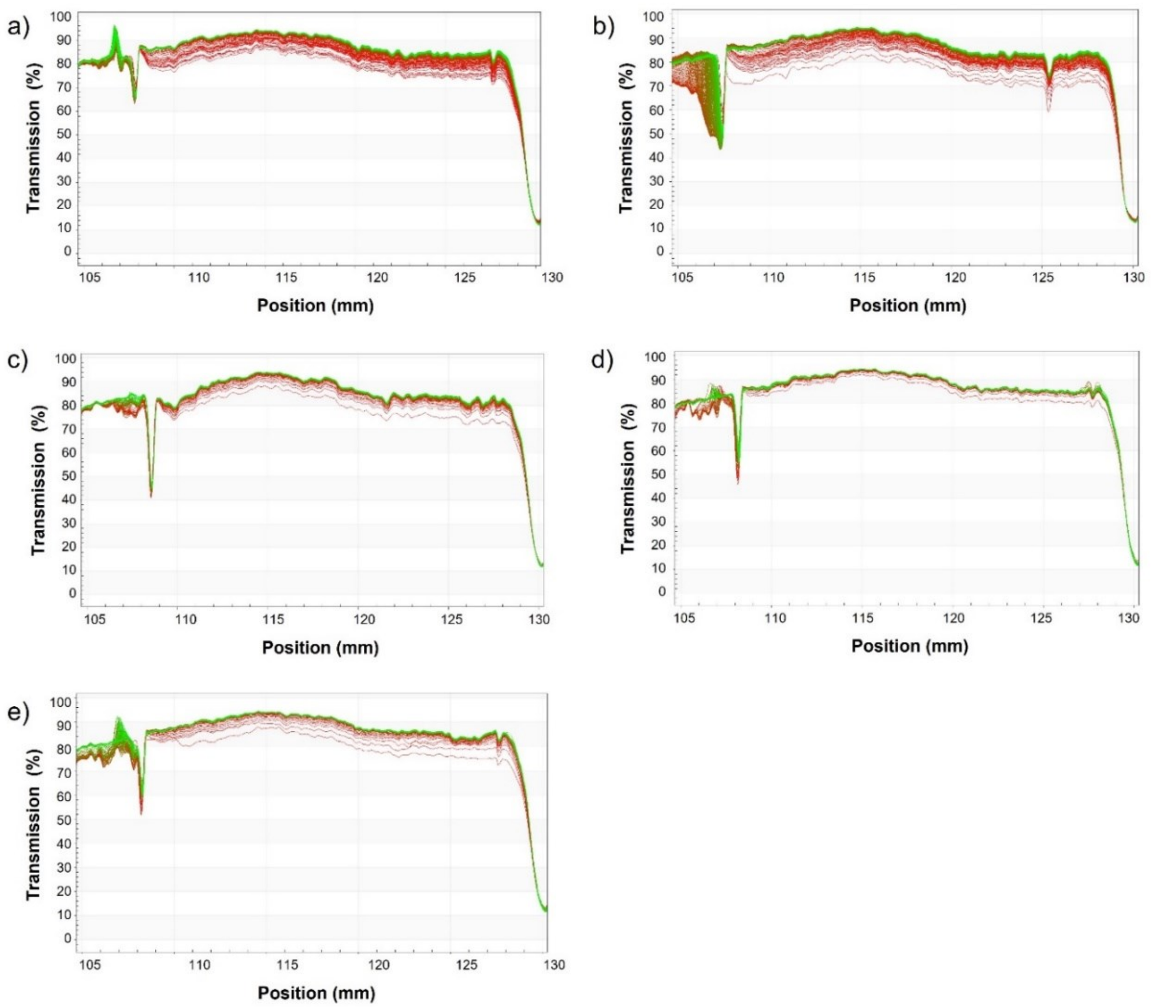

Figure 4. Transmittance profiles of a) soil:water extract without NPs, b) Ag NPs at 100 mg·L^{-1} in soil:water, c) AgHEC NPs at 100 mg·L^{-1} in soil:water extract, d) AgHEC NPs at 10 mg·L^{-1} in soil:water extract, e) AgHEC NPs at 1 mg·L^{-1} in soil:water extract.

To conclude, because of the LoD of DLS, ELS and CSA techniques, colloidal characterization of NPs at the lowest investigated concentrations (1 and 10 mg·L^{-1}) did not provide any results, confirming that colloidal characterization of Ag at the concentrations released from Ag-WDs cannot be performed using these techniques.

Appendix SI3. Electron Dispersive X-ray analysis

From EDX analysis of PVA-Ag alternative, both Ag and Cl were detected before (Figure 5b) and after immersion (Figure 5d) in synthetic sweat, suggesting the presence of AgCl on the surface of the fibres, probably as residues of Ag NPs synthesis.

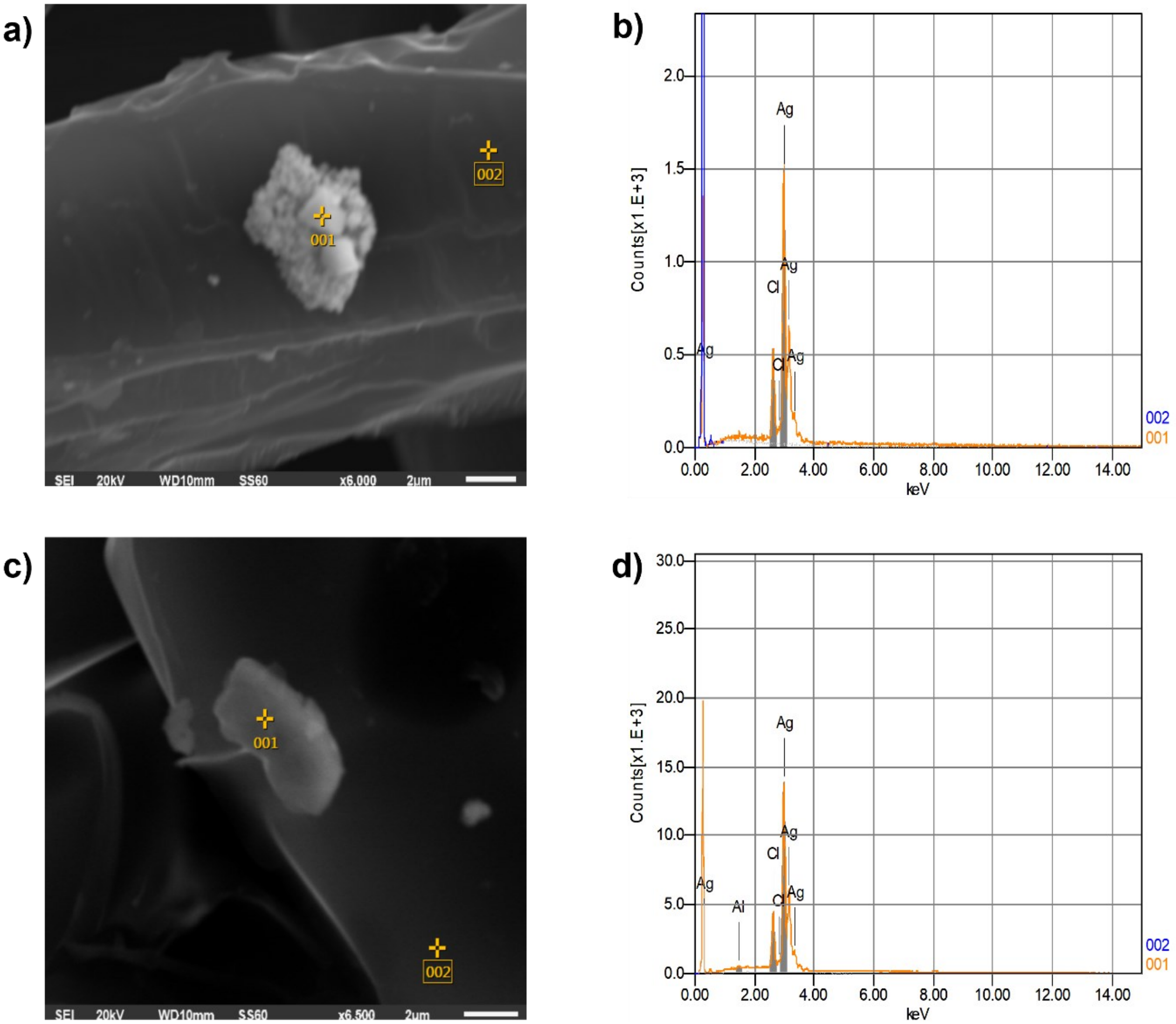

Figure 5. SEM images of PVA-Ag a) before and c) after immersion, and EDX spectrum b) before and d) after 24h of immersion in synthetic sweat.

Appendix SI4. Results of the analysis performed considering the SbD citeria

Table SI4. Results of the analysis performed to evaluate the SbD alternatives and commercial Ag-WDs against each SbD criterion.

Criteria	Analysis	Investigated parameters / units	Tested conditions	SbD alternatives Ag-WDs					Commercial Ag-WDs	
				PLLA-Ag	PLLA-AgHEC	PVA- Ag	PVA-AgHEC.1h	PVA-AgHEC.2h	Acticoat Flex3	Acticoat Flex 7
Mechanical strength	SEM analysis	Fibres size (nm) Mean ± SD	Before immersion	2480±484	70 ± 23	15369 ± 1270	16363 ± 1764	16210 ± 1492	6790 ± 1875	9831 ± 1564
			After immersion	2710 ± 345	N.A.	16378 ± 2133	15470 ± 1770	16320 ± 1480	7684 ± 773	7877 ± 1336
		Fibres shape	Before immersion	Uniformly distributed	Uniformly distributed	Uniformly distributed	Uniformly distributed	Uniformly distributed	Uniformly distributed	Uniformly distributed
			After immersion	Preserved	Not preserved*	Preserved	Preserved	Preserved	Preserved	Preserved
Antimicrobial efficacy	Bacterial reduction	%	-	97		89	100	100	-	-
Leaching of Ag from Ag-WDs immersed in	Ag released-immersion in AFW	(μgWD^{-1}) Mean ± SD	1 day	0.79±0.04		9.1±0.7	7±2	10.9±0.8	231±3	253±27
			3 days	0.93±0.03		10±1	8±1	12.84±0.05	228.9±0.2	265±6

**environmental media**			7 days	0.02±0.03		6.3±0.5	7.8±0.8	15.01±0.3	276±7	319±25
			14 days	1.44±0.05		13±1	7.1±0.5	14.35±0.07	254±10	297±28
			21 days	1.0±0.3		11.7±0.3	6±1	13.1±0.2	298±9	355±28
			28 days	2.85±0.08		23.5±3.1	7.0±0.9	13.8±0.9	330±3	382±20
	Ag released-immersion in AMW	(μgWD^{-1}) Mean ± SD	1 day	4.3±0.4		8.7±0.7	10±3	34±9	188±7	194±1
			3 days	14±1		21±3	13±4	42±9	195±6	206±1
			7 days	28.8±2.5		15±4	16±4	55±11	221±6	229±7
			14 days	49±2		32±6	20±5	66±13	225±5	226±2
			21 days	48.9±0.4		11.5±0.7	17±5	64±10	216±1	225±6
			28 days	57.65±0.04		40±5	21±5	67±15	228.1±0.5	239±9
	Ag released-immersion in soil:water extract	(μgWD^{-1}) Mean ± SD	1 day	4.9±0.6		28.7±2.6	11±4	26±1	4889±9	3769±2
			3 days	2.7±0.1		15±4	8±4	29±1	6182±12	6046±3
			7 days	5.2±2.5		17±2	10±4	30±1	7108±11	7382±6
			14 days	3.6±0.8		18±4	11±5	34.2±0.6	8126±17	8456±2
			21 days	3±1		12±3	11±7	26.52±0.03	9236±9	9450±3

			28 days	3±1		14±4	12±7	23.4±0.5	9776±6	10073±4
**Cost-Effectiveness**	Total Ag content	(µgWD^{-1}) Mean ± SD	-	1912±148		124±25	41±7	89±14	27872±1029	31943±1124
	Ag released-immersion in sweat	(µgWD^{-1}) Mean ± SD	1 day	1.6±0.1		8.5±0.7	10±1	28±4	68.8±0.5	64.5±2.0
			3 days	3.5±0.3		16±3	12.3±0.7	32±4	69±2	64.6±0.3
			7 days	8.0±1.5		30±6	18±2	46±6	76±2	77.7±0.6
	Total Ag content VS Ag released-immersion in sweat	%	1 day	0.1		6.9	26.4	26.9	0.2	0.2
			3 days	0.2		12.7	33.8	31.1	0.2	0.2
			7 days	0.4		24.2	49.0	45.0	0.3	0.2
